# Modulation of GPC-4 and GPLD1 serum levels by improving glycemic indices in type 2 diabetes: Resistance training and *hawthorn* extract intervention

**DOI:** 10.1016/j.heliyon.2023.e15537

**Published:** 2023-04-15

**Authors:** Ali Heidarianpour, Maryam Keshvari, Siamak Shahidi, Mohammad Zarei

**Affiliations:** a Bu- Ali Sina University, Faculty of Sport Sciences, Hamedan, Iran; b Department of Physiology, School of Medicine, Hamadan University of Medical Sciences, Hamadan, Iran; c Neurophysiology Research Center, Hamadan University of Medical Sciences, Hamadan, Iran

**Keywords:** Diabetes, Exercise training, Medicinal plants, GPC-4, GPLD1, Insulin

## Abstract

**Aims:**

This study was designed to investigate the effects of resistance training (RT) and *hawthorn* extract (Ha) on Glypican-4 (GPC-4) and Insulin-regulated glycosylphosphatidylinositol-specific phospholipase D (GPLD1) serum levels in T2DM and to examine the relationship of these variables with glycemic indexes.

**Method:**

40 male Wistar rats were divided into five equal groups: Healthy Control (H-C), Diabetes Control (D-C), Diabetes Resistance training (D-RT), Diabetes *Hawthorn* (D-Ha), and Diabetes Resistance training *Hawthorn* (D-RT-Ha). T2DM was induced with a 4-week high-fat diet (HFD) and one dose of STZ intraperitoneal injection (35 mg/kg). 1-week after the injection, RT (with a range of 50%–100%1RM/3 day/week) and gavage of Ha extract (100 mg/kg/day) was performed for 12 weeks.

**Results:**

The glycemic indices improvement (reducing blood glucose and increasing serum insulin level) caused by RT and/or Ha increased GPC-4 and decreased GPLD1 in the T2DM rats, but these positive changes were more effective in the combination of RT + Ha. A strong correlation was also observed between GPC-4 and GPLD1 with blood glucose and insulin.

**Conclusion:**

The increase in serum GPC-4 levels was probably due to the direct effect of RT + Ha, and the improvement of glycemic indexes after RT and Ha. The double effect of RT + Ha can be a regulatory mechanism for GPC-4 and its related factors in controlling T2DM complications.

## Graphical abstract

**Figure:**
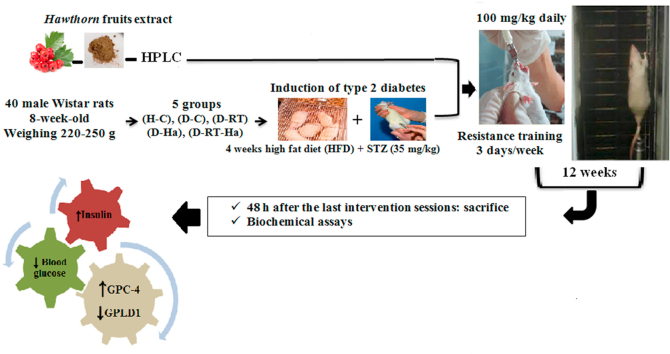

## 1 Introduction

Diabetes mellitus is one of the most common metabolic disorders that occurs when the body is unable to properly manage insulin secretion, insulin action, or both, and is characterized by chronic hyperglycemia [1]. Type 1 diabetes mellitus (T1DM) accounts for 5–10% of people with diabetes [2]. This type of diabetes leads to a severe insulin deficiency by destroying pancreatic beta cell autoimmunity [1]. Type 2 diabetes mellitus (T2DM) is the most common type of diabetes in adults, accounting for approximately 90–95% of all diabetics worldwide [2], and with the lack of sensitivity to insulin due to insulin resistance, a decrease in insulin production and finally dysfunction of pancreatic beta cells appear [1].

Glypican-4 (GPC-4) is a well-known adipokine that increases insulin hormone signaling by interacting with the insulin receptor [3]. It has been reported that GPC-4 serum levels have a positive correlation with body fat levels and insulin resistance (IR). Hence, serum level of this adipokine was introduced not only as a marker for body mass index (BMI) but also as an independent marker for IR [4]. Zhu et al. (2014) showed that fasting insulin (FIns) and homeostasis model assessment of insulin resistance (HOMA-IR) are the two factors that have the greatest effect on GPC-4 serum level [5].

It has been reported that serum levels of GPC-4 increase in pre-diabetic subjects with impaired glucose tolerance, but decrease in newly diagnosed T2DM patients [6]. In fact, serum GPC-4 levels display a bell-shaped profile from healthy subjects to pre-diabetic subjects and then to diabetic patients. In line with the reduction of GPC-4, the relative losses of T2DM increase. Hence, GPC-4 may be involved in the development of T2DM. A short-term increase in glucose or insulin (for example, in the oral glucose tolerance test: OGTT) may have a direct effect on the release of GPC-4 [7].

Insulin-regulated glycosylphosphatidylinositol-specific phospholipase D (GPLD1) is the most likely GPC-4 cleavage option, whose gene is, in turn, regulated by insulin. Therefore, the increase in insulin in the first stages of obesity can increase the breakdown of GPC-4, the consequence of which is an increase in GPC-4 serum levels. As the disease progresses, the increase in GPLD1 may be part of a compensatory response to increased insulin demand [8]. On the other hand, it has been reported that increasing IR in GPLD1 producing cells can decrease the activity of this enzyme and decrease the serum level of GPC-4; thus, the insulin sensitivity (IS) is further reduced and the progress of the disease accelerates [4]. Therefore, maintaining GPC-4 serum levels in people with IR or diabetes mellitus can reduce their need for insulin therapy [9].

The role of herbs with hypoglycemic properties in the treatment of diabetics cannot be ignored [10,11]. *Hawthorn* is one of the oldest medicinal plants and its fruits and leaves have medicinal value with favorable therapeutic effects for gastrointestinal diseases, fat metabolic disorders, cardiovascular diseases and other disorders [12,13]. Although it has been used in treating diseases for over 2,000 years, a limited number of studies have examined its pharmacological mechanisms. *Hawthorn* has been reported to contain flavonoids, flavone C-glycosides, catechin, amines, trypropene saponins, and oligomeric procyanidin, of which flavonoids are the major biologically active compounds [14]. It has been shown that *hawthorn* extract has a protective effect against diabetic complications through antioxidant activity, hypoglycemia, hyperlipidemia and the ability to normalize insulin secretion [2]. Overall, the basic mechanisms of *hawthorn* extract against diabetes may be related to inhibiting intestinal α-glycosidase, reducing hepatic gluconeogenesis, improving lipid metabolism, and restoring insulin sensitivity [2]. As previous studies reported, *hawthorn* inhibits pancreatic lipase and pancreatic alpha-amylase enzymes [15]. Clinically, *hawthorn* may be one of the valuable medicinal herbs in the future showing a dual effect against hyperglycemia and hyperlipidemia in human type 2 diabetic patients [2]. Although experimental studies have reported positive effects of this plant and its family plants, in none of them have the simultaneous effects on blood sugar, insulin, adipokine levels of GPC-4 and GPLD1 been investigated.

Numerous studies have demonstrated the usefulness of exercise in managing T2DM [16]. Among the numerous health benefits of physical activity, one of the most important ones is related to its role in improving total glucose homeostasis [17]. In addition to controlling blood glucose, exercise provides benefits such as reducing IR and improving aerobic capacity, muscle strength, body composition and endothelial function [18].

Part of this improvement may be due to change of adipokine profile following exercise activity. Thus, the beneficial effects of exercise on metabolic diseases have been linked to its effect on the activity of adipokine [19]. Many studies have been conducted to investigate aerobic training in diabetes [[20], [21], [22]]. Aerobic training that involves large groups of muscles includes exercise such as walking, cycling, swimming and running [23]. However, 80% of people with T2DM are overweight or obese, and many have movement problems, peripheral neuropathy, visual impairment, or cardiovascular disease. For this population, achieving the required volume and intensity of aerobic exercise may not be easy and resistance training may be more effective. Resistance training uses muscle strength to move a weight or work against a resistance load that causes brief and isolated muscle group activity [24]. It has attracted increasing attention in the last decade [25,26].

In particular, resistance training with its insulin-sensitizing effect releases the brake on beta-oxidation and helps improve metabolic flexibility and more balanced use of fatty acids as substrates. Therefore, increased insulin sensitivity may contribute to increased clearance of lipids from the blood. Increased insulin receptor protein expression in response to resistance exercise may be another important adaptation responsible for the insulin-sensitizing effect of exercise [27].

Also, skeletal muscle is responsible for about 80% of insulin-mediated glucose uptake in the postprandial state, and uptake is significantly reduced in people with T2DM. In fact, compared to lean healthy subjects, skeletal muscle of people with T2DM shows a reduced capacity to oxidize glucose and fat [28]. Considering that chronic resistance training that increases muscle mass and strength is mainly due to the induction of muscle hypertrophy and neuromuscular regeneration [28], we believed that there is a possibility that by supplementing antioxidants along with exercise, insulin function and its downstream factors can be affected. Since no research has yet investigated the effect of resistance training with *hawthorn* extract on the levels of glycemic indices, GPLD1 and GPC-4 in type 2 diabetic rats induced by high fat diet-streptozotocin (HFD -STZ), this study was designed.

## 2 Materials and methods

### 2.1 Animals

**Fig. 1:**
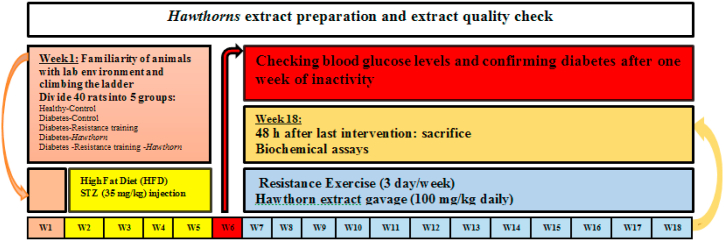
Outline of the implementation of the study protocol.

In this study, forty 6-week-old male Wistar rats weighing 180–200 gr were purchased as a research sample from Hamadan University of Medical Sciences. All animals were kept in the Rodent Exercise Physiology Lab of Bu Ali Sina University at 22±1 °C, 12-h light-dark cycle, and in transparent polycarbonate cages. Before starting the research protocol, the rats were familiarized with the lab environment and resistance exercises (climbing the ladder) for a week. After the familiarization period, the rats were divided into 5 equal groups: Healthy Control (H-C), Diabetes Control (D-C), Diabetes Resistance training (D-RT), Diabetes *Hawthorn* (D-Ha), and Diabetes Resistance training *Hawthorn* (D-RT-Ha). All experiments of this study were performed according to the instructions of the Ethics Committee for Lab Animals of Hamadan University of Medical Sciences with code IR.UMSHA.REC.1400.202. Fig. 1 shows an outline of the implementation of the study protocol. Briefly, before starting the research, a familiarization course with the laboratory environment and resistance training was conducted for a week. Then, after grouping, a four-week HFD period was started. After the HFD period and confirmation of obesity with Lee's index, STZ injection was performed. One week after STZ injection and confirmation of diabetes induction, resistance exercise protocol and hawthorn extract gavage was performed for 12 weeks.

### 2.2 Induction of type 2 diabetes mellitus

HFD and STZ were used to induce T2DM [[29], [30], [31]]. After a one-week familiarization phase of the rats with the lab environment and resistance training, the rats in the diabetic group were fed a HFD for 4 weeks, which included 58% fat calories, 27.5% carbohydrate calories, and 14.5% protein calories. The total caloric content of fatty foods was ∼4900 kcal/kg, which was prepared for weekly consumption and kept at a temperature −4 °C. To prepare a HFD with these characteristics, we used animal butter and added supplements to prevent the reduction of protein, casein and the amino acid methionine, and to prevent the reduction of minerals and vitamins. Percent by weight: 33.3% Carbohydrate+ 32.3% Fat+ 17.5% Protein+ 5.7% Vitamin and mineral mix+ 3.9% Fiber+ 7.1% Water = 100% Total (see Table 1). Normal food powder (prepared by Pars Animal Feed Company) with a total caloric content of ∼3160 kcal/kg was also used for the H-C group. Percent by weight: 57% Carbohydrate+ 2% Fat+ 17.5% Protein+ 4.9% Vitamin and mineral mix+ 6.6% Fiber+ 12% Water = 100% Total (see Table 1) [[29], [30], [31]]. After 4 weeks of HFD, Lee index was used to assess rat obesity according to the formula [Lee Index (weight (g 0.33) ÷ Nasoanal length *(*mm))] [29]. An index greater than 0.3 was considered obese. Then, to induce T2DM, STZ (35 mg/kg) dissolved in citrate buffer (0.1 M, pH = 4.5) was injected subcutaneously. 3–7 days after STZ injection, glucose levels were measured from the animal's tail blood sample and FBG levels above 300 mg/dL were considered as a criterion for diabetes. In addition, citrate buffer was injected intraperitoneally to the rats of the control group. It is noteworthy that after induction of diabetes, all rats used Normal Pellet Diet (ND). A high-fat diet (HFD) was only able to induce type 2 diabetes.

**Table 1:** Composition of the normal pellet and the high fat Diets.

	Normal Pellet Diet (ND)		High Fat Diet (HFD)	
	Weight %	Calorie%	Weight %	Calorie%
Carbohydrate	57	72.1	33.3	27.5
Fat	2	5.6	32.3	58
Protein	17.5	22.1	17.5	14.5
Vitamin and mineral mix	4.9	–	5.7	–
Fiber	6.6	–	3.9	–
Water	12	–	7.1	–
Total	100	100	100	100

### 2.3 Preparation and dosage of *hawthorn* hydro-alcoholic extract

*Hawthorn* fruits were collected from the Zagros Mountains in western Iran and identified and approved in the herbarium of Bu Ali Sina University with the code 8034. To prepare the hydro-alcoholic extract, the *hawthorn* seeds were removed and the fruit flesh was incubated for 24 h to dry. After drying, the fruits were pulverized with an electric grinder. We poured 100 g of the powder into the Erlenmeyer flask and add 80% ethanol solvent, then blocked the Erlenmeyer flask with a parafilm to prevent the solvent from evaporating. It was then smoothed using Whatman paper. These steps were repeated 3 times to ensure that all fruit compounds were dissolved in the desired solvent. An incubator at 47 °C was used to evaporate the excess ethanol. The remaining extract, in the form of a semi-solid orange mass, was poured into glass containers, covered with aluminum foil, and stored in the refrigerator and away from light until use. *Hawthorn* extract compositions were separated by Reverse Phase HPLC Method, Using an Agilent system 1260 equipped with a waters RP C18 column (4.6 × 250 mm, 5 μm of particle size, Waters, USA) Diode Array detector (DAD, G4212B). The samples were washed with a gradient system consisting of solvent A (0.1% formic acid) and solvent B (acetonitrile) at a flow rate of 1.0 ml/min. The column temperature was maintained at 30 °C and the injection volume was 10 μl. After gradient elution, the compounds were determined by comparing the storage time of the standards with DAD detection at 280, 300 and 340 nm, respectively. The contents of each compound were estimated based on the peak level and calibration curves of the relevant standards.

To prepare a 5-mg/ml extract, the dried material was dissolved in 0.9% NaCl with pH 7.4. One week after STZ injection and confirmation of diabetes induction, *hawthorn* extract was gavaged to D-Ha and D-RT-Ha rats at a dose of 100 mg/kg daily for 12 weeks at the beginning of the resistance training period. Previous studies have shown the effectiveness of this dose [[32], [33], [34]]. Also, rats in the control group were gavage with 100 mg/kg of water daily for 12 weeks.

### 2.4 Resistance training

Resistance training included climbing on a ladder with a weight hanging from the animal's tail. The rats were instructed on how to climb the ladder before induction of diabetes. One week after STZ injection and confirmation of diabetes induction, resistance training was performed 3 days/week for 12 weeks. To determine one-repetition maximum (1RM), 50% of the animal's weight was considered for the first attempt. When the animal reached the top of the ladder, 20 g of weight was added in the next attempt to reach the top of the ladder. If the rat was able to carry a weight, another 20 g would be added to the previous weight in the next attempt. This training continued until it was done 10 times or the rat fell down the ladder. The resting interval between repetitions was 2 min. The protocol for weeks 1–12 was as follows: weeks 1–3 with 50%, weeks 4–6 with 70%, weeks 7–9 with 80%, and for the remaining sessions 100% of 1RM was considered. Warming and cooling in this protocol included going up and down the ladder twice in each session for each rat [16].

### 2.5 Biochemical assays

48 h after the last training session and consumption of *hawthorn* extract, all rats were anesthetized by intraperitoneal injection of ketamine (50 mg/kg) and xylazine (4 mg/kg) [35] after 12 h of fasting. After anesthesia, their abdominal cavity was opened with scissors and blood samples were taken from inferior vena cava.

At this stage, FBG of rats was measured with a glucometer (Acuu-Chek Active, Roche, Germany). Then, blood samples were centrifuged at 3000 rmp for 10 min. After serum separation, serum samples were placed in a 1.5 cc microtype; and were kept at −35 °C for biochemical measurements. Using the ELISA sandwich method, serum of FIns (Mercodia Rat Insulin ELISA Kit, Uppsala, Sweden), GPLD1 (ELISA Kit, ZellBio GmbH, Ulm, Germany) and GPC-4 (ELISA Kit, ZellBio GmbH, Ulm, Germany) was measured in the ELISA reader (BioTek-ELx808) according to the manufacturer's guidelines with 450 nm wavelength. The HOMA-IR and homeostasis model index of insulin release measurement (HOMA-IS) were calculated based on the formula HOMA-IR= (FIns, μU/ml) × (FBG, mmol/L)/22.5 and HOMA-IS = (20 × FIns (μU/ml))/(FBG (mmol/L) - 3.5) [7].

### 2.6 Statistical analyses

After collecting data, descriptive and inferential statistics were used for data analysis. Shapiro-Wilk test was used for testing normal data distribution and Levin's test was used to determine the homogeneity of variances. One-way analysis of variance (Tukey's post hoc test) was used to examine the mean differences between the groups. In addition, Pearson correlation test was used to examine the relationship between the variables. All results are shown as Mean ± SEM and P < 0.05 values were considered as statistically significant. SPSS 22 was used for statistical analysis and Prism GraphPad 8 was used for drawing graphs.

## 3 Results

### 3.1 The compositions of the hawthorn extract by HPLC

**Fig. 2:**
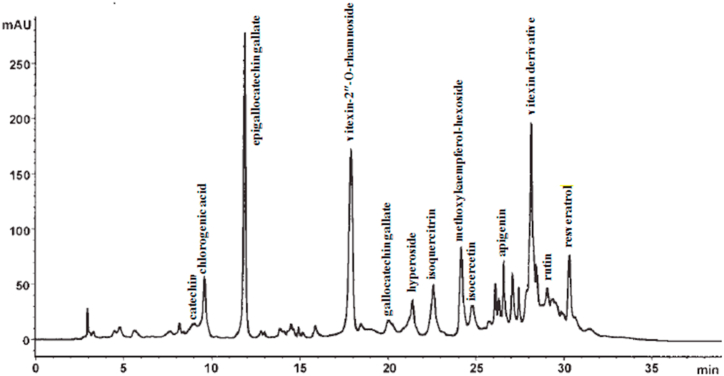
The compositions of *hawthorn* extract by HPLC. The calibration range of each compound was as follows: catechin (0.16–36.6 μg), chlorogenic acid (0.75–9.80 μg), epigallocatechin gallate (0.21–32.76 μg), rutin (0.62–9.70 μg), vitexin (0.37–5.95 μg), isocercetin (0.66–16.40 μg), quercetin (0.11–11.40 μg), methoxykaempferol-hexoside (0.87–5.74 μg), apigenin (0.18–8.0 μg), gallocatechin gallate (0.14–4.87 μg), vitexin-2″-*O*-rhamnoside (0.84–19.45 μg), isoquercitrin (0.54–6.98 μg), hyperoside (0.43–8.23 μg), and resveratrol (0.85–25.8 μg).

The calibration range of each compound was as follows: catechin (0.16–36.6 μg), chlorogenic acid (0.75–9.80 μg), epigallocatechin gallate (0.21–32.76 μg), rutin (0.62–9.70 μg), vitexin (0.37–5.95 μg), isocercetin (0.66–16.40 μg), quercetin (0.11–11.40 μg), methoxykaempferol-hexoside (0.87–5.74 μg), apigenin (0.18–8.0 μg), gallocatechin gallate (0.14–4.87 μg), vitexin-2″-*O*-rhamnoside (0.84–19.45 μg), isoquercitrin (0.54–6.98 μg), hyperoside (0.43–8.23 μg), and resveratrol (0.85–25.8 μg). Fig. 2 shows the compositions of the *hawthorn* extract by HPLC.

### 3.2 T2DM induction confirmation

The mean FBG and body weight (BW) of rats at the beginning of the study were (6.68 ± 0.091 mmol/L) and (186.685 ± 4.35 gr), respectively. Induction of T2DM was performed using four weeks of HFD and STZ injection. Mean FBG levels (12.20 ± 0.15 mmol/L) and BW (348.56 ± 0.68 gr) of the rats increased significantly compared to the baseline after 4 weeks of HFD. After confirming the obesity of the rats based on Lee index, STZ-injection was performed. One week after the injection, FBG was found to be higher compared to the HFD stage (22.27 ± 0.194 mmol/L). These results confirm the induction of T2DM. Table 2 shows the Mean ± SEM of fasting levels of blood glucose (FBG), fasting insulin (FIns), body weight (BW) and Lee index at the beginning of the protocol, four weeks after HFD, and one week after STZ injection, and 12 weeks after the research protocol.

**Table 2:** Mean ± SEM of FBG, FIns, BW and Lee index in the stages pre and post induction of type 2 diabetes.

	Group	Pre HFD	Post HFD	Post HFD/STZ	Post protocol
FBG (mmol/L)	H-C	6.71 ± .205	6.70 ± .154	6.96 ± .181	6.78 ± .314
	D-C	6.83 ± .237	11.73 ± .231	21.76 ± .351	22.98 ± .155
	D-RT	6.55 ± .222	12.63 ± .236	22.94 ± .124	18.09 ± .197
	D-Ha	6.56 ± .198	12.41 ± .250	22.18 ± .407	17.96 ± .379
	D-RT-Ha	6.74 ± .243	12.04 ± .364	22.21 ± .464	15.43 ± .778
FIns (μU/ml)	H-C	10.44 ± .282	10.62 ± .449	10.36 ± .335	10.25 ± .282
	D-C	10.61 ± .355	13.94 ± .407	7.35 ± .138	4.98 ± .122
	D-RT	11.01 ± .266	13.85 ± .757	6.74 ± .345	7.80 ± .252
	D-Ha	10.76 ± .332	14.74 ± .791	6.65 ± .230	7.67 ± .360
	D-RT-Ha	10.86 ± .223	14.10 ± .631	7.19 ± .169	9.85 ± .615
BW (gr)	H-C	185.27 ± 2.12	215.50 ± 1.70	216.50 ± 1.70	268.37 ± 2.35
	D-C	188.30 ± 1.67	349.50 ± 1.19	344.25 ± 1.49	332 ± 2.85
	D-RT	186.62 ± 2.56	348 ± 1.29	343.25 ± 1.49	339 ± .577
	D-Ha	185.82 ± 2.63	347 ± 1.91	342.25 ± 1.10	339 ± .707
	D-RT-Ha	187.40 ± 2.69	349.75 ± .946	344.75 ± 1.03	324.25 ± 3.32
Lee Index	H-C	.029 ± .001	.027 ± .001	.027 ± .001	.026 ± .001
	D-C	.029 ± .000	.032 ± .000	.031 ± .000	.029 ± .000
	D-RT	.0291 ± .000	.031 ± .000	.030 ± .000	.029 ± .000
	D-Ha	.029 ± .001	.031 ± .001	.031 ± .001	.029 ± .000
	D-RT-Ha	.029 ± .000	.031 ± .000	.031 ± .000	.028 ± .000

### 3.3 The improvement of glycemic index caused by resistance training + *hawthorn* increased the GPC-4 and GPLD1 levels in T2DM

**Fig. 3:**
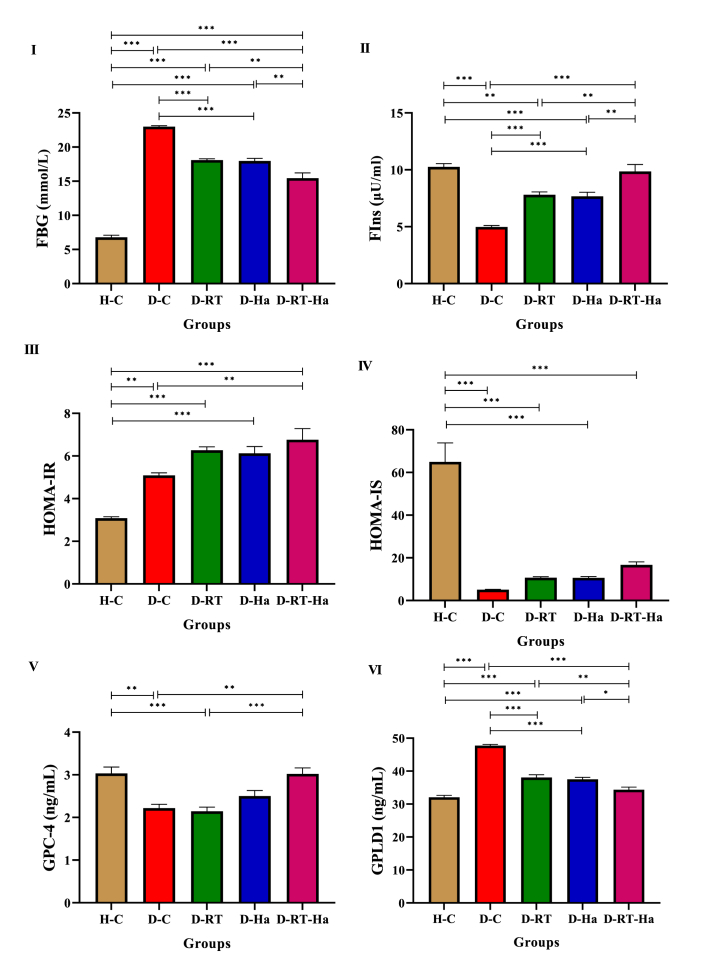
RT + Ha improved glycemic index and increased serum GPC-4 levels. Changes of FBG (I), FIns (II), HOMA-IR (III), HOMA-IS (IV), GPC-4 (V), and GPLD1 (VI) in diabetic rats following 12 weeks of resistance training and *hawthorn* extract treatment. * Significantly different (P < 0.05), ** significantly different (P < 0.01), *** significantly different (P < 0.001). (ANOVA and subsequent Tukey's HSD, P < 0.05).

To evaluate the effect of resistance training and *hawthorn* extract on glycemic index, FBG and serum FIns levels were measured. Also, HOMA-IR and HOMA-IS were calculated. Twelve weeks after *hawthorn* consumption and resistance training, significant changes in FBG (Fig. 3I) (F = 194.396, P = 0.001) and Fins (Fig. 3II) (F = 33.236, P = 0.001) levels were observed. In addition, changes in FBG and FIns caused differences in HOMA-IR (Fig. 3III) (F = 25.746, P = 0.001) and HOMA-IS (Fig. 3IV) (F = 37.206, P = 0.001) between the study groups. Our results showed that diabetes caused an increase in FBG and a decrease in serum FIns levels in D-C rats compared to the H-C group (P = 0.001), such that 12 weeks of resistance training and *hawthorn* intervention alone and together caused the adjustment of these variables in comparison with D-C (P = 0.001). Comparison of three D-RT, D-Ha and D-RT-Ha groups also shows the effectiveness of *hawthorn* combination and resistance training in lowering FBG and increasing FIns compared to the single implementation of these interventions in D-RT groups (PFBG = 0.004, PFIns = 0.009) and D-Ha (PFBG = 0.006, PFIns = 0.006). Following an increase in FBG and a decrease in FIns in diabetic rats, we saw an increase in HOMA-IR (P = 0.002) and a decrease in HOMA-IS (P = 0.001) in the D-C group compared to the H-C group. 12 weeks of resistance training and *hawthorn* intervention together in D-RT-Ha caused the adjustment of HOMA-IR in comparison with D-C (P = 0.007). In addition, there was no significant difference in HOMA-IR and HOMA-IS between D-RT, D-Ha and D-RT-Ha groups.

In this study, there was a significant difference in the GPC-4 (Fig. 3V) (F = 11.815, P = 0.001) and GPLD1 (Fig. 3VI) (F = 83.527, P = 0.001) levels of the study groups.

Induction of T2DM decreased GPC-4 and GPLD1 levels. The level of GPC-4 after 12 weeks in the D-RT-Ha group significantly increased compared to the D-C (P = 0.003), and D-RT (P = 0.001) groups. The increase in GPC-4 level in D-Ha (P = 0.056) and D-RT-Ha (P = 1.000) groups was so great that no difference was found between these groups and the H-C group.

We saw a significant decrease in GPLD1 level after 12 weeks in the D-RT, D-Ha, and D-RT-Ha groups compared to the D-C group (P = 0.001). In addition, this decrease was higher in the D-RT-Ha group compared to the D-Ha (P = 0.026) and D-RT (P = 0.008) groups. There was no significant difference in GPLD1 level between D-RT and D-Ha groups. The decrease in GPLD1 level in D-RT-Ha group was not different from that of the H-C group.

### 3.4 The correlation of GPC-4 and GPLD1 with FBG, FIns, HOMA-IR, HOMA-IS

As shown in Table 3, based on Pearson's correlation test, GPC-4 had a very high positive relationship with FIns level (P = 0.001, r = +0.702), and a very high negative relationship with FBG level (P = 0.001, r = −0.702). Also, GPC-4 had a positive and moderate relationship with HOMA-IS (p = 0.013, r = +0.542), but no association with HOMA-IR.

**Table 3:** The correlation of GPC-4 and GPLD1 with FBG, FIns, HOMA-IR, and HOMA-IS.

		FBG	FIns	HOMA-IR	HOMA-IS	GPC-4	GPLD1
**GPC-4**	Pearson Correlation	−.704^a^	.702^a^	−.270	.542^b^	1	−.614^a^
	Sig. (2-tailed)	.001	.001	.250	.013		.004
**GPLD1**	Pearson Correlation	.837^a^	−.895^a^	.146	−.628^a^	−.614^a^	1
	Sig. (2-tailed)	.001	.001	.539	.003	.004	

a Correlation is significant at the 0.01 level (2-tailed).

b Correlation is significant at the 0.05 level (2-tailed).

In addition to GPC-4, GPLD1 had a very high negative relationship with FIns level (p = 0.001, r = −0.895) and a positive relationship with FBG level (p = 0.001, r = +0.837). However, GPLD1 had a negative and moderate relationship with HOMA-IS (p = 0.003, r = −0.628), but no association with HOMA-IR.

The results of Pearson's test for GPC-4 and GPLD1 showed a moderate negative relationship between these two variables (P = 0.004, r = −0.614).

## 4 Discussion

In this study, diabetes increased FBG and decreased the FIns level and, following these changes, HOMA-IR increased and HOMA-IS decreased in diabetic rats. 12 weeks of *hawthorn* consumption and resistance training caused significant changes in FBG, FIns, HOMA-IR, and HOMA-IS. Comparing the groups, we saw the effectiveness of the combination of resistance training and *hawthorn* in reducing FBG and increasing FIns compared to the separate implementation of these interventions.

12 weeks of resistance training and *hawthorn* intervention together caused the adjustment of HOMA-IR. In addition, the results of HOMA-IS showed that resistance training and *hawthorn* together increased the function of β pancreatic cells in rats though this increase was not statistically significant. Exercise can improve insulin release by increasing the function and mass of pancreatic β cells in diabetic rats [36]. In fact, exercise increases insulin release from each pancreatic islet and prevents the discharge of insulin accumulation in them. Following these changes, exercise releases compensatory insulin and prevents hyperglycemia. It has been reported that the improvement in pancreatic cell function resulting from exercise in the first stage is related to insulin release, such that in type 2 diabetic rats this stage of insulin release is impaired [37]. Other reasons for improving blood sugar control following resistance training include an increase in lean body mass and internal muscle changes [27]. In confirmation of these reasons, Holten et al. (2004) showed that six weeks of resistance training on one foot increased insulin function by increasing the content of glucose transporter type 4 (GLUT4) protein, insulin receptor, protein kinase B-***α***/***β*** and glycogen synthase while the untrained foot did not change [38]. Therefore, in the process of increasing blood sugar control, the amount of insulin needed to clear a certain amount of glucose decreases [27]. On the other hand, *hawthorn* extract also reduced FBG and increased FIns in diabetic rats. The flavonoids in *hawthorn* cause hypoglycemia. The hypoglycemic effect of flavonoids can be the result of increased hepatic hexokinase and glucokinase activity, protection and even increased beta cell density in islets of Langerhans, increased expression of activated glucose carriers, Glucose 6 phosphatase or increased glucose uptake by liver cells, fat and muscle [39,40]. Diabetes is associated with adverse changes in lipid profile, plasma insulin levels, blood glucose levels and also an increase in oxidative stress indices [41]. Quercetin, as a bioflavonoid in *hawthorn*, has anti-inflammatory, antioxidant and anti-apoptotic effects [42]. Quercetin activates the antioxidant enzyme glutathione peroxide and prevents oxidative damage. In addition, quercetin reduces oxygen-glucose deficiency. Reduction of neurotoxicity due to free radicals and oxytocin is another function of quercetin in *hawthorn* and prevents DNA strand breakage and cytotoxicity [42]. In this study, *hawthorn* consumption decreased FBG and increased FIns in diabetic rats, but this result was more significant in combination with resistance training.

Probably, resistance training and *hawthorn* together improve blood glucose and insulin function through AMPK. AMPK is a critical node that links lipid signaling and metabolism and is a positive regulator of IR. Decreased AMPK activity, characterized by its phosphorylation level, reflects the potential negative effects of lipid concentrations on insulin signaling. *Hawthorn* and exercise training significantly increase AMPK phosphorylation and not only improve lipid metabolism, but also improve glucose uptake in skeletal muscles. PPARγ in *hawthorn* and exercise have been shown to increase GLUT4 gene expression or plasma membrane translocation and help improve glucose homeostasis [[43], [44], [45]]. Our study showed that resistance training and *hawthorn* probably affected glucose homeostasis by increasing the expression of GLUT4 in skeletal muscles and improved muscle glucose absorption.

Another result of this study was the decrease in the levels of GPC-4 and GPLD1 in diabetic rats, where 12 weeks of resistance training and *hawthorn* together (D-RT-Ha group) caused a further increase in these variables. The increase was so great that there was no difference compared to the H-C group.

One of the reasons for changes in serum GPC-4 levels is the interaction of this adipokine with the insulin and its direct relationship with insulin signaling [9]. Our results showed a very high positive relationship between GPC-4 and levels of FIns, and a very high negative relationship between GPC-4 and FBG levels. We saw a decrease in blood sugar, an increase in insulin and GPC-4 in a D-RT-Ha group while Ussar et al. (2012) showed that levels of this adipokine in the bloodstream of rodents and humans are positively correlated with body fat levels and insulin receptor.

They also showed that GPC-4, unlike other insulin sensitizers, enhances insulin signaling through direct interaction with the insulin receptor. On the other hand, they showed that GPC-4 increases the differentiation of adipocytes. By depleting GPC-4, they found that insulin receptor activation and adipocyte differentiation decreased.

Although at first glance the bioactivity of GPC-4 as a booster of insulin receptor seems to be heterogeneous with increasing serum levels in people with IR, they showed that elevated serum GPC-4 levels could be a new regulatory mechanism which responds to IR by adipose tissue [4]. Lee et al. (2014) found that in addition to adipose tissue, mRNA and GPC-4 protein are also expressed in skeletal muscle. Therefore, both tissues seem to play an important role in GPC-4 system levels. They also showed that the circulating GPC-4 increased in pre-diabetic patients with impaired glucose tolerance but decreased in new patients with T2DM [7]. In fact, serum levels of GPC-4 show a bell-shaped profile from healthy subjects to pre-diabetic subjects and then to diabetic subjects. As GPC-4 decreases, relative T2DM losses increase. Therefore, GPC-4 depletion may be involved in T2DM proliferation [7].

Based on the findings of this study, resistance training and *hawthorn* alone and together decreased the serum level of GPLD1 in diabetic rats. Down-regulation of GPLD1 was associated with increased serum insulin level. So far, the effect mechanisms of resistance training and *hawthorn* extract on GPLD1 serum level have not been investigated. Nevertheless, studies have shown that GPLD1 serum level can be regulated by various factors such as insulin serum level, hyperglycemia, ROS and inflammation [46,47].

Based on the strong and inverse relationship between insulin and GPLD1 in this study, it seems that the serum level of insulin can be an effective construct in the serum level of GPLD1. Deeg et al. (2001) showed that diabetes increases the expression of GPLD1 in the liver; and insulin therapy decreases GPLD1 mRNA and GPLD1 serum levels in non-obese diabetic rats [47]. Thus, by inhibiting the hepatic synthesis of GPLD1, insulin reduces the serum levels of GPLD1 in diabetic patients and diabetic rats [48]. Additionally, it has been suggested that the increase in GPLD1 could be part of a compensatory response to increased insulin demand [8]. Hence, the increase in insulin serum level in rats of resistance training and *hawthorn* intervention groups partially reduced the need for this compensatory response through GPLD1. In addition, the direct correlation between serum levels of GPLD1 and FBS shows that the serum level of GPLD1 can be regulated by blood glucose levels. In line with this finding, various studies have shown that hyperglycemia increases the serum level of GPLD1 [[47], [48], [49]]. Deeg et al. (2001) found that increasing hyperglycemia by 2–5 times more than normal levels increases the serum activity of GPLD1 and liver mRNA of this enzyme in non-obese diabetic rats and STZ-induced diabetic CD-1 rats [47]. In addition, it has been shown that incubation of isolated rat islets and βTC3 cells with high glucose increases cell activity and GPLD1 mRNA. These findings indicate that glucose and especially hyperglycemia play an important role in the synthesis and release of GPLD1 [47].

Therefore, the improvement caused by resistance training and *hawthorn* extract in rat's glycemic status can reduce the serum level of GPLD1. In addition, a laboratory study has shown that by increasing oxidation stress, H2O2 causes an increase in mRNA GPLD1 in the field of macrophage cells [46].

Therefore, the antioxidant effects of resistance training and *hawthorn* extract can be the possible mechanism involved in reducing the serum levels of GPLD1 diabetic rats. Another explanation for reducing the serum level of GPLD1 can be the anti-inflammatory effects of resistance training and *hawthorn* extract. GPLD1 can hydrate GPI anchors in a number of inflammatory membrane proteins (such as CD55, CD59, and CD106), and its hydrolyte products can lead to increased adjustment in inflammatory cytokines including IL-1 and TNF-α [50]. Therefore, the anti-inflammatory nature of the resistance training and *hawthorn* extract can at least reduce the serum level of GPLD1. The correlation between the serum surfaces of the GPLD1 and the antioxidant and anti-inflammatory structures require further research. Obviously, this research can reveal amazing mechanisms involved in antioxidant and anti-inflammatory effects of resistance training and *hawthorn* extract and their correlation with GPLD1.

*Hawthorn* extract along with resistance training in this study increased the serum level of GPC-4. Liu et al. (2014) showed that PPARγ is activated by rosiglitazone (ROSI) - a family of thiazolidinediones and PPARγ agonists - and causes a significant increase in mRNA and GPC-4 protein in subcutaneous adipose tissue; however, no significant difference was observed in the expression of mRNA and GPC-4 protein in the visceral adipose tissue [51]. Therefore, the level of GPC-4 mRNA and protein expression in subcutaneous adipose tissue seems to be associated with PPARγ activation [51]. In the study by Ussar et al. also, the drug troglitazone - another member of the thiazolidinediones family and the PPARγ agonist - increased the differentiation of control preadipocyte cells; however, it had no significant effect on GPC-4 deficient cells [4]. Hence, the association between GPC-4 and the PPARγ nuclear receptor becomes more apparent. Considering the fact that PPARγ has a role in macronutrient metabolism, it has become an important research and clinical trend. This receptor is the target of insulin-sensitizing drugs - thiazolidinediones - in the treatment of T2DM [52]. Specifically, the rutin and quercetin [53] in *hawthorn* are effective in controlling blood glucose and trigger the expression of the PPARγ gene. This gene is the target receptor for ROSI. ROSI enhances insulin receptor in different cell types [54]. Due to the reasons mentioned above, resistance training and *hawthorn* together have positive effects on improving glycemic index and serum GPC-4 levels in diabetics. Thus, resistance training and *hawthorn* reached a common point in lowering FBG, increasing FIns, and increasing GPC-4 from different path ways and this synergy was more effective for diabetic rats. One of the limitations of this study is not investigating the downstream factors of insulin signaling in female rats, and it has not been done in any study so far, and researchers are suggested to investigate the effect of resistance training and *hawthorn* on GPLD1 and GPC-4 in female rats in the future.

## 5 Conclusion

The results of this study revealed that GPLD1 serum levels decrease after resistance training and *hawthorn* hydroethanolic extract; this reduction can be due to the improvement of serum glycemic and insulin profile. It is possible that the increase in insulin and the decrease in blood glucose prevent the increase in compensatory response of GPLD1 to the demand for insulin. Increasing insulin and modulating GPLD1 enzyme activity due to resistance training and *hawthorn* hydroethanolic extract increased GPC-4 and insulin sensitivity in diabetic rats. More research is needed to better understand the biological behavior of GPC-4 and GPLD1 in response to resistance training and herbal therapy.

## Abbreviations

Fasting blood glucose (FBG), Fasting insulin (FIns), High Fat Diet (HFD), Glucose transporter type 4 (GLUT4), Glypican-4 (GPC-4), Insulin-regulated glycosylphosphatidylinositol-specific phospholipase D (GPLD1), *Hawthorn* (Ha), Homeostasis model assessment of insulin resistance (HOMA-IR), Homeostasis model assessment of insulin secretion (HOMA-IS), Insulin resistance (IR), Insulin sensitivity (IS), Resistance training (RT), Rosiglitazone (ROSI), Streptozotocin (STZ), Type 2 diabetes mellitus (T2DM).

## Author contribution statement

Heidarianpour Ali: Conceived and designed the experiments; Wrote the paper.

Keshvari Maryam: Performed the experiments; Wrote the paper.

Shahidi Siamak, Zarei Mohammad: Analyzed and interpreted the data; Wrote the paper.

## Funding statement

Authors would like to thank the Deputy of Research andTechnology, Hamadan University of Medical Sciences, forits support for the study [IR.UMSHA.REC.1400.202/grantnumber 14005124066].

## Data availability statement

Data will be made available on request.

## Declaration of interest's statement

The authors declare no conflict of interest.
